# An evaluation by dental clinicians of cutting characteristics and haptic perceptions in 3D‐printed typodont teeth: A pilot study

**DOI:** 10.1002/jdd.13749

**Published:** 2024-10-23

**Authors:** Alexander J. Cresswell‐Boyes, Graham R. Davis, Asa H. Barber, Mahentha Krishnamoorthy, Swati R. Nehete

**Affiliations:** ^1^ Dental Physical Sciences Unit, Centre for Oral Bioengineering, Institute of Dentistry, Barts and the London School of Medicine and Dentistry, Queen Mary University of London London UK; ^2^ Peninsula Dental School, Faculty of Health, University of Plymouth Plymouth UK; ^3^ School of Engineering, London South Bank University London UK; ^4^ Oral Health Innovation, GlaxoSmithKline Consumer Healthcare, Haleon Weybridge UK; ^5^ Centre for Teaching and Innovation, Institute of Dentistry, Barts and the London School of Medicine and Dentistry, Queen Mary University of London London UK

**Keywords:** dental education, 3D printing, simulation‐based medical education, haptic perception, preclinical training, operative

## Abstract

**Objectives:**

This study aimed to compare the haptic perception of clinicians to the cutting response of 3D‐printed typodont teeth and commercial typodont teeth and human extracted teeth.

**Methods:**

Twenty clinicians were asked to perform a Class I cavity preparation on commercial typodont teeth, 3D‐printed typodont teeth, and human extracted teeth, while the forces were recorded via a three‐axis load cell. The haptic perception of clinicians was also evaluated through a response questionnaire comparing commercial and 3D‐printed typodont teeth.

**Results:**

The study found that clinicians used similar forces (*p* = 0.53) to cut both the 3D‐printed typodont teeth (1.37 N) and the human extracted teeth (1.44 N), but more force was needed to cut the commercial typodont teeth (3.71 N). The response questionnaire indicated that clinicians rated the 3D‐printed typodont teeth highly compared to the commercial teeth. The 3D‐printed dentine received favorable feedback from clinicians, and the 3D‐printed enamel was rated higher compared to the commercial equivalents.

**Conclusions:**

The results of the study suggest that 3D‐printed typodont teeth offer a comparable haptic perception to human extracted teeth and can be used as an effective tool for preclinical dental learning. Moreover, the study highlights the advantages of 3D‐printed typodont teeth over commercial typodont teeth in terms of haptic perception.

## 1 INTRODUCTION

Simulation‐based medical education (SBME) is a well‐established method used in medical and dental fields to ensure students and clinicians acquire the necessary skills for safe clinical practice.^1^ SBME allows participants to practice procedural skills in a safe and realistic environment before active clinical care.^2^, ^3^ Traditionally, extracted teeth were used exclusively for dental education, but with a decline in tooth extractions and limitations in using extracted teeth,^4^, ^5^, ^6^, ^7^, ^8^ dental schools have turned to artificial teeth (typodont teeth) mounted in a simulated head (phantom head) for preclinical learning.^9^, ^10^, ^11^, ^12^, ^13^

However, drawbacks to using typodont teeth have been reported, including lack of realism, low abrasion resistance, and increased force required for cutting, which reduces the accuracy of the operative experience for students.^9^, ^12^, ^14^, ^15^, ^16^, ^17^ Haptic perception, the ability for a user to experience a tactile feeling or a sense of touch when operating an instrument,^18^ is something that traditional typodont teeth lack.^14^, ^15^ In the United Kingdom, dental schools extensively use both extracted teeth and typodont teeth in their teaching. As an alternative, 3D printing has emerged as a viable option for creating dental training models due to its advantages, such as designing teeth based on patient anatomy and using multimaterials to replicate enamel and dentine.^19^, ^20^, ^21^, ^22^, ^23^

Literature on the use of 3D printing in SBME is growing, and some studies have shown promising results. For example, Reymus et al.^22^ developed a workflow for dental educational institutions with access to cone beam computed tomography and 3D printing facilities to create resin teeth for endodontic teaching purposes. Students in this study rated the 3D‐printed teeth higher for availability, fairness due to standardization, comfort in practicing endodontics, and hygiene compared to extracted teeth. However, the study was limited by the lack of data on cutting perception, anatomical accuracy, and preference for 3D‐printed teeth compared to the current artificial teeth used in their school.

Other studies, such as the one by Hanafi et al.,^19^ have developed modular 3D‐printed dental training models for endodontic training using cone beam computed tomography data from a human skull. The resultant 3D‐printed models and extracted teeth were used by students to perform root canal treatment, and the treatments were evaluated to be acceptable. Students' perceptions of the models were highly rated, with 96% indicating better preparation for clinical situations and recommending the use of the models in preclinical training and teaching. The authors emphasized the importance of student perception and stakeholder involvement in evaluating 3D‐printed models.

In addition, Cresswell‐Boyes et al.^15^ conducted a study comparing the haptic perception of cutting human extracted teeth and 3D‐printed typodont teeth using a multiple‐parameter variable of material elastic modulus and hardness. Results indicated that the 3D‐printed teeth demonstrated comparable mechanical properties to human extracted teeth under cutting conditions. However, the perception of using such teeth by students and clinicians is lacking.

This study aims to build on the work previously published^15^, ^24^ and to evaluate the drilling perception and experience of these 3D‐printed typodont teeth from a clinical dental educator's point of view. To fulfil this aim, two studies were conducted:

Study 1: Evaluate forces clinicians apply when using a dental handpiece to cut extracted, commercial and 3D‐printed typodont teeth.

Study 2: Obtain and review feedback from clinicians using a questionnaire on the use of commercial and 3D‐printed typodont teeth. Clinicians’ feedback on cutting response will be compared with the forces recorded in Study 1.

## 2 MATERIALS AND METHODS

### 2.1 Ethical approval and data protection

Ethical approval for the study was obtained from the Queen Mary Research Ethics Committee (QMERC20.586), with the feedback being collected anonymously using the Online Surveys (formerly BOS; JISC, UK) platform. The clinical dental educators (40 participants) consented before participation and were informed that their participation would be anonymized. Data were collected, processed, and stored following data protection laws.

### 2.2 Participant recruitment

In this study, participant recruitment was conducted via an open call for volunteers among experienced dental clinical educators at Queen Mary University of London, without specific inclusion or exclusion criteria based on years of practice or other experience‐related factors. The aim was to include a diverse group of clinicians with varied practice backgrounds to gather a broad spectrum of feedback on the haptic perceptions and cutting characteristics of the 3D‐printed typodont teeth.

For Study 1, a group of 20 clinicians was randomly selected to create cavities while their applied forces were recorded. In Study 2, a set of 20 different clinicians was randomly assigned to provide qualitative feedback on the cutting response, ensuring they had no prior exposure to the force measurement process. This random coupling minimized bias and enhanced the reliability and validity of the findings by maintaining the independence of each study component.

### 2.3 Specimen selection

A variety of tooth samples were examined in this study and were aggregated into three groupings:

Group 1. Anonymized extracted teeth obtained from a human tissue bank; with ethical approval obtained from Queen Mary Research Ethics Committee (QMREC2011/99). Used in Study 1 only.
Group 2. Commercially available typodont teeth (ANA‐4 Z, Frasaco GmbH). Used in both Studies 1 and 2.
Group 3. 3D‐printed typodont teeth were developed in this work using a composite materials approach. Used in both Studies 1 and 2.

**FIGURE 1:**
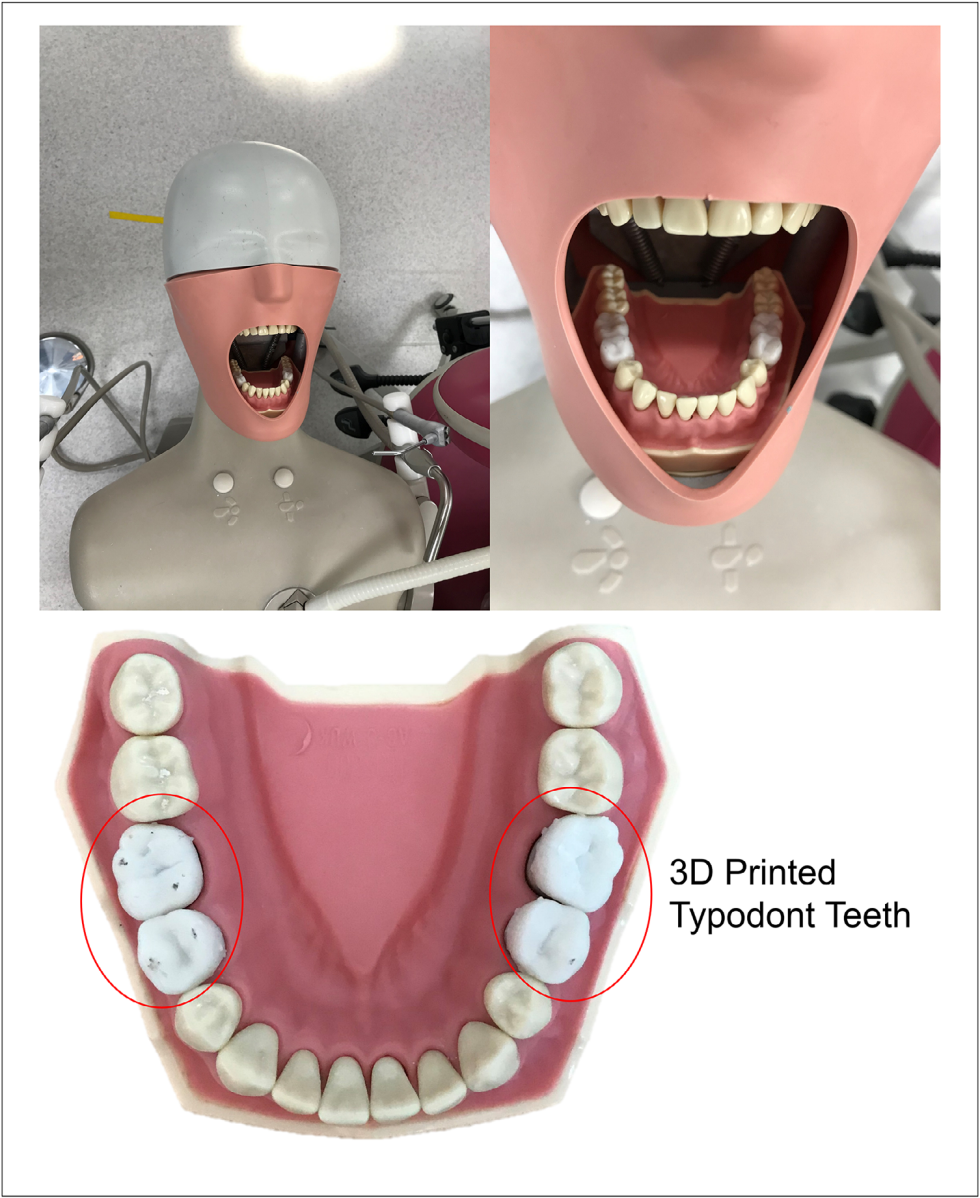
Positions of the 3D‐printed typodont teeth within the Frasaco mandibular plate, including other Frasaco typodont teeth mounted within a phantom head.

A recent study^15^ found that commercially available typodonts used in Group 2 required 30% higher cutting forces compared to extracted dentine and 122% higher cutting forces compared to enamel. However, among the tested commercial typodont teeth, Frasaco teeth were found to best resemble extracted tissue and ranked highly. Notably, students at Queen Mary University of London typically use Frasaco teeth for practice operative procedures, mounted to a dental arch (ANA‐4, Frasaco GmBH) within a phantom head mannequin (A Dec Inc.) equipped with a turbine unit and computer access (Figure 1).

### 2.4 X‐ray microtomography

**FIGURE 2:**
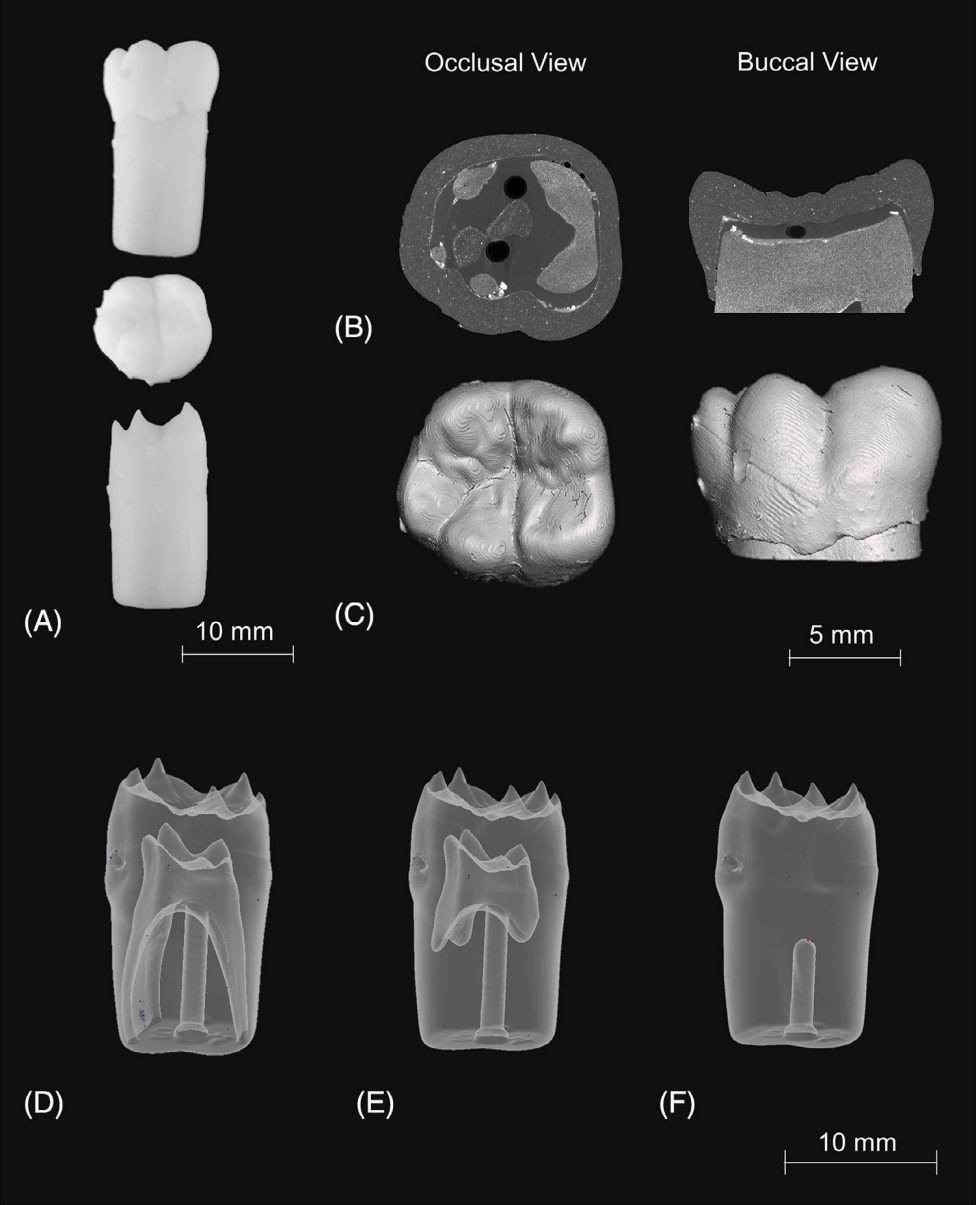
(A) Images of the 3D‐printed mandibular first molar. (B) Reconstructed X‐ray microtomography (XMT) images of the 3D‐printed typodont tooth. (C) 3D renderings of the 3D‐printed typodont tooth. Reconstructed XMT images of 3D‐printed dentine from the mandibular first molar, showing (D) a fully formed pulp, complete with root canals, (E) a semi‐formed pulp, with limited root canals, and (F) no pulp cavity.

The geometry of the Frasaco typodont teeth was obtained using the MuCAT2 X‐ray microtomography (XMT) scanner^25^ and converted into *.stl file format.^24^ After 3D printing, the typodont teeth were imaged at 15‐µm voxel size with 40 kV and 405 µA to ensure the geometry matched that of the extracted tooth (Figure 2).

### 2.5 Production of the 3D‐printed typodont teeth

**FIGURE 3:**
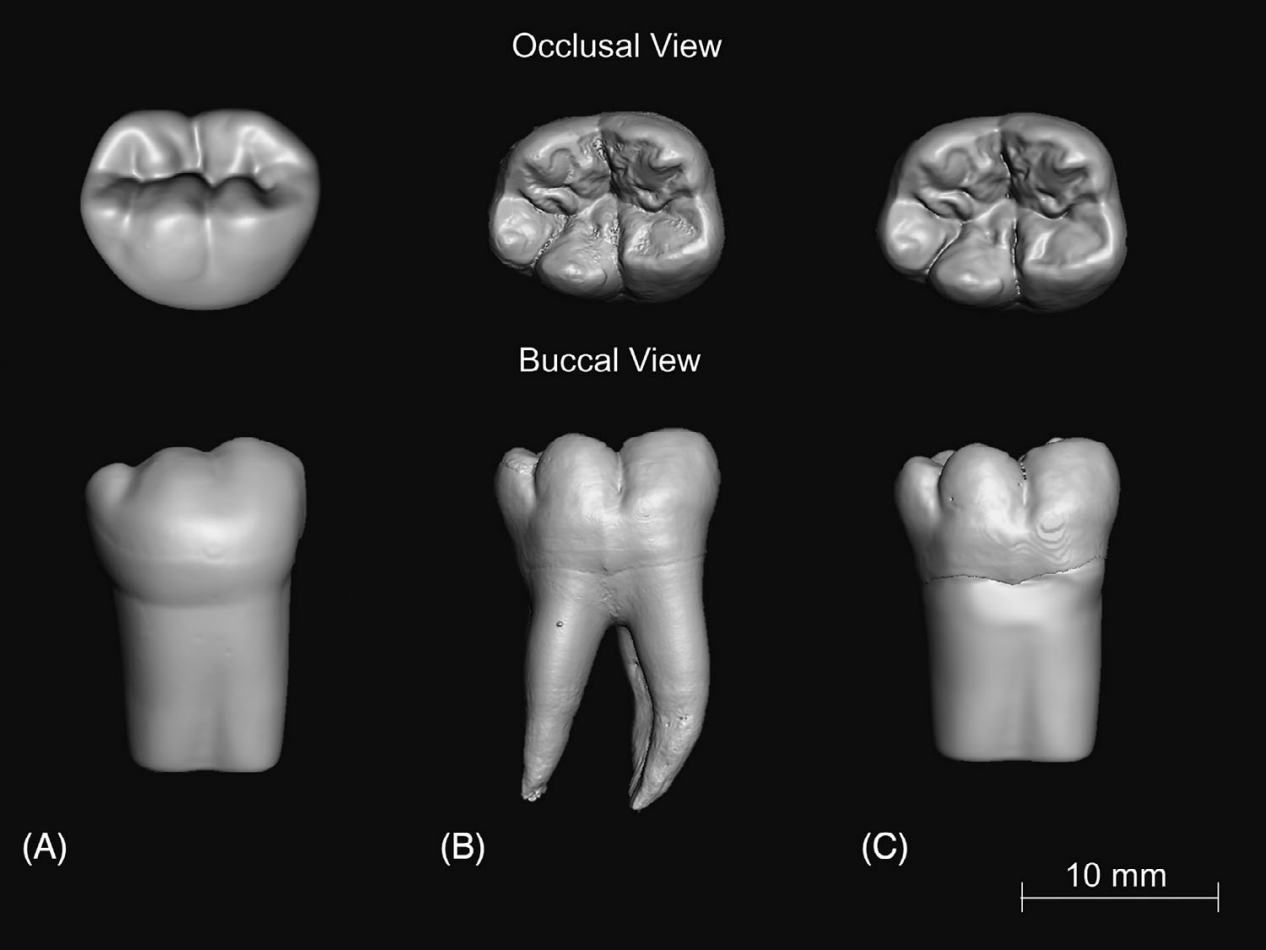
The geometry of the 3D‐printed typodont tooth was designed using the jaw mounting from a Frasaco typodont tooth and the crown of an extracted mandibular first molar. 3D renderings from reconstructed XMT images of (A) Frasaco typodont tooth, (B) extracted mandibular first molar, and (C) 3D‐printed typodont tooth developed in this study.

Production of the 3D‐printed typodont teeth followed the protocols outlined in Cresswell‐Boyes et al.^15^ and Cresswell‐Boyes et al.^24^ The design of the 3D‐printed typodont teeth was created by merging the scanned occlusal and internal geometry of the extracted molar with the Frasaco base to allow the mounting of the 3D‐printed typodont tooth to the Frasaco typodont jaw (Figure 3).

For the typodont model, separate mixtures were made for enamel and dentine components. The enamel mixture consisted of 20 wt.% carbonated hydroxyapatite (CHAp), while the dentine mixture consisted of 5 wt.% hydroxyapatite (HAp). The CHAp was prepared as outlined by Landi et al.^26^ and the HAp used was Capital® Sintered HAp (Plasma Biotel Ltd.). The powders were ground and sieved (below <38 µm) before being added to a photopolymerizable polymer resin (Anycubic 405 nm Rapid Resin, Anycubic) as a weight percentage. The mixture was mechanically mixed for 24 h at 37°C to ensure complete dispersion. Each material was printed separately using an Anycubic Photon (Anycubic), with a layer height of 50 µm and a layer cure time of 25 s. Once printed, the models were washed in ethanol to remove the uncured resin. The enamel was then fixed to the dentine with an uncured enamel mixture and cured with a handheld curing light (EliparTM DeepCure‐S, 3M). The enamel structure was printed 2% larger for easy fitting. The entire typodont was further cured using a Formlabs Cure (Formlabs Inc.). Finally, ribbon wax (Metrodent) was melted and injected into the screw opening of the 3D‐printed typodont to fill up the pulp cavity. [Correction added on December 06, 2024, after first online publication: The methodology numbers have been corrected in this section.]

### 2.6 Force measurements—Study 1

**FIGURE 4:**
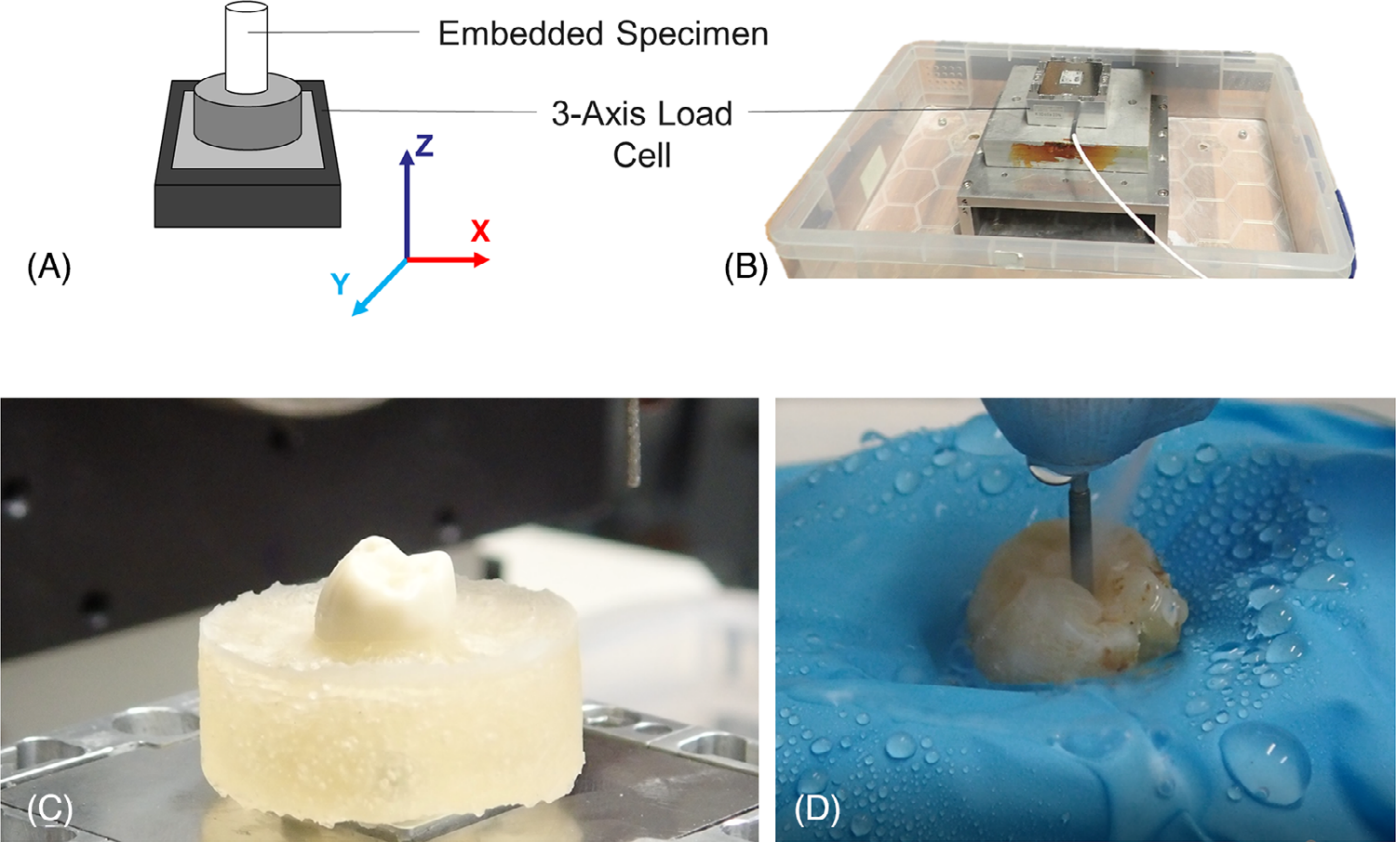
The experimental setup used in Study 1 for measuring the forces required to cut the specimens. (A) A schematic diagram of an embedded specimen mounted on a three‐axis load cell. (B) A photograph of the three‐axis load cell housed in a container to provide a drainage system. (C) A photograph of an embedded Frasaco typodont tooth mounted on a three‐axis load cell, before the addition of the rubber dam. (D) A photograph of a human extracted tooth being cut by a participant, with the three‐axis load cell positioned underneath the rubber dam to prevent damage from irrigation.

In Study 1, 20 dentists from the Queen Mary University of London and general practice participated in a cavity preparation experiment. Participants were recruited, and their informed consent was obtained. Class I cavities with a depth of 2.00 mm (Black 1904) were prepared on extracted, commercial (Frasaco), and 3D‐printed teeth. Mandibular first molars were embedded in acrylic blocks (Kemdent Simplex Rapid, Associated Dental Products Ltd. [Figure 4C]) and mounted to a three‐axis load cell (Model 3A60A, Interface Force Measurements Ltd.). A high‐speed dental handpiece (TE‐95 BC Alegra Dental Air Rotor Handpiece, The W&H Group) with two different diamond rotary instruments (straight fissure and inverted cone) was used, chosen at the discretion of the participants. Diamond rotary instruments were changed between each test and participants. The different types of teeth were presented to the participants blind, and in random order. Participants were given 15 min between each tooth, to rest and prevent lethargy from affecting the results. The participants were not given a time limit and were instructed to avoid touching the load cell to prevent interference with the data recording. Plastic sheeting (rubber dam) and a drainage system were used to keep the load cell dry (Figure 4D). This set‐up closely followed that outlined in Cresswell‐Boyes et al.^15^ (Figure 4). The cavity preparation process was recorded using a high‐definition camera with an LED light attachment. Data and video were synchronized at the beginning and end of preparation. Load data was recorded and logged using a signal amplifier (ME‐Meβsysteme GmbH) and software (GSVmulti Version 1.40, 2018; ME‐Meβsysteme GmbH) that recorded real‐time load data in three directions: mesiodistal (X), buccolingual (Y), and occlusal (Z).

### 2.7 Questionnaire design and implementation—Study 2

In Study 2, 20 clinically qualified dental educators from the Queen Mary University of London participated in an evaluation study. Participants from Study 1 were not recruited for Study 2, to ensure the typodont teeth were evaluated without previous knowledge. Clinicians were given a Frasaco mandible plate containing four 3D‐printed typodont teeth (second premolars and first molars) and 12 Frasaco typodont teeth, which were mounted into a phantom head (Figure 1). The clinicians were instructed to cut and drill two of each type of typodont tooth, with the 3D‐printed typodont teeth presented on the left and right sides based on their dominant hand. Questionnaires (Table 1) with closed and open questions, including numerical response scale questions, were provided after the participation with the 3D‐printed typodont teeth. The questionnaire had been tested before and after a clinical skills session with experienced clinicians and was adapted for this study using the 3D‐printed typodont teeth.

**TABLE 1:** The 10‐question survey given, to compare the participants’ cutting perception of the 3D‐printed teeth compared with the extracted and Frasaco teeth.

Typodont questionnaire									
*Please circle a response for each question (Questions 1–6) on a scale of 1–10 (1 = unlike, 10 = identical)*.									
1.	Please rate the **occlusal surface** detail in comparison to an extracted tooth.								
1	2	3	4	5	6	7	8	9	10
2.	Please rate the **likeness of drilling the enamel** (haptic perception) in comparison to an extracted tooth.								
1	2	3	4	5	6	7	8	9	10
3.	Please rate the **likeness of drilling the dentine** (haptic perception) in comparison to an extracted tooth.								
1	2	3	4	5	6	7	8	9	10
4.	Please rate the **likeness of exposing the pulp** (haptic perception) in comparison to an extracted tooth.								
1	2	3	4	5	6	7	8	9	10
5.	Please rate the 3D printed typodont for its **overall value in operative experience and training** in comparison to an extracted tooth.								
1	2	3	4	5	6	7	8	9	10
6.	Please rate the 3D printed typodont for its **overall value in operative experience and training** in comparison to commercial typodont teeth.								
1	2	3	4	5	6	7	8	9	10
7.	Would you use these models again?								
Yes					No				
8.	What aspect of the model was your **least favorite**?								
9.	What aspect of the model was your **most favorite**?								
10.	Any other comments?								

The survey included numerical response scale questions, asking the participants to rate between 1 and 10 with 1 being unlike and 10 being identical against extracted and commercial typodont teeth.

### 2.8 Statistical analysis

Statistical analysis was carried out using SPSS statistics software (Version 29.0, 2022; SPSS Inc.). Significant differences between results were calculated through a one‐way analysis of variance (ANOVA) for the parametric data collected in Study 1, and the Mann–Whitney *U* test for the nonparametric data collected in Study 2. Values *p* < 0.05 were considered significantly different.

## 3 RESULTS

**FIGURE 5:**
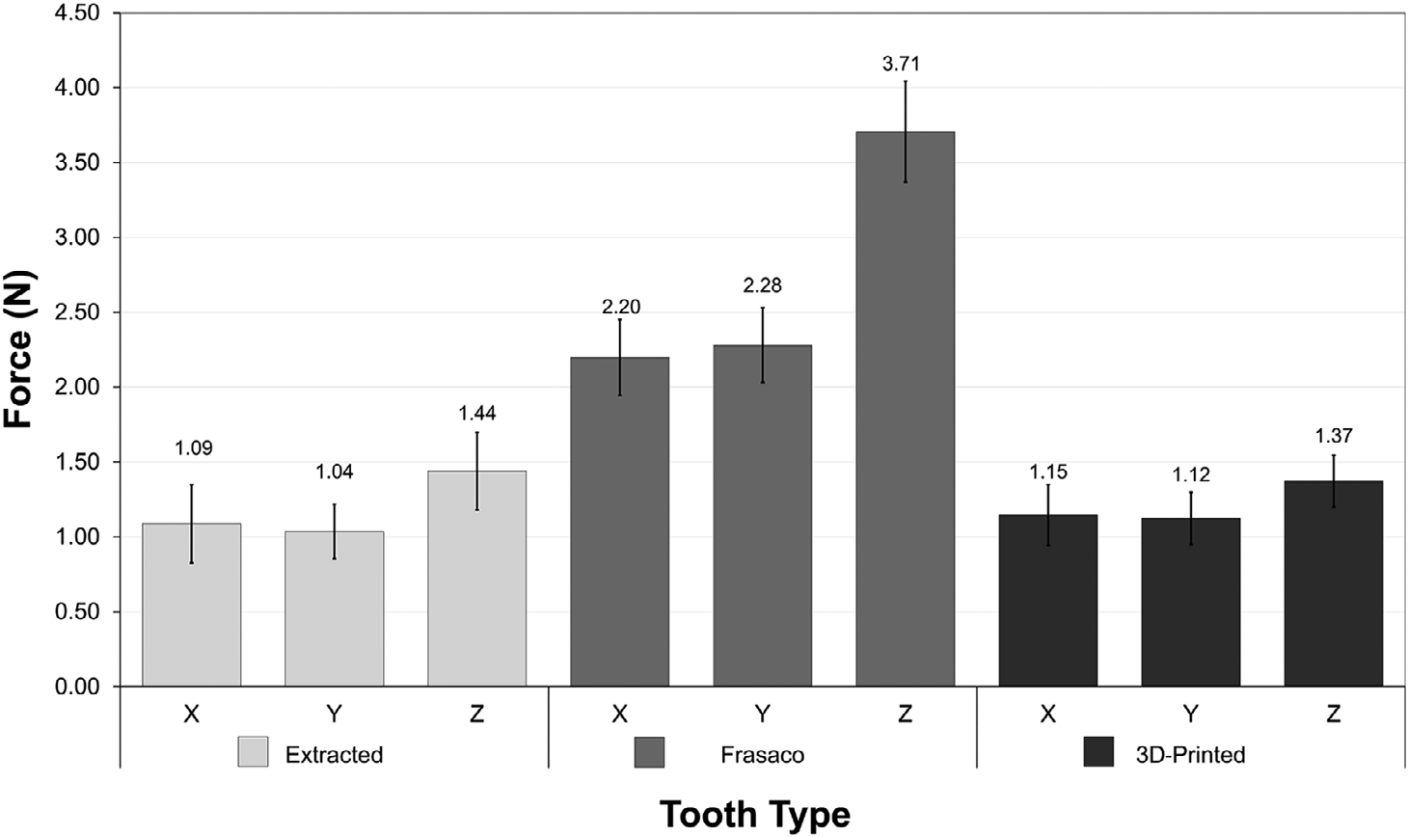
Mean force used by clinicians to cut the 3D‐printed typodont teeth, Study 1. Extracted mandibular molars and artificial mandibular molars from Frasaco were used as comparisons. Directions of cut were defined as; mesiodistal (*X*), buccolingual (*Y*), and occlusal (*Z*). Error bars are presented as the standard deviation (SD) of the sample (*n *= 20).

The mean force used to cut 3D‐printed typodonts from Study 1 is shown in Figure 5. These force results showed a similar trend seen in Cresswell‐Boyes et al.,^15^ where more force was required to cut commercial typodont teeth compared to human extracted teeth, in all directions. In the mesiodistal direction, the clinicians recorded forces of 1.09 N (±0.26) for extracted, 2.20 N (±0.25) for commercial (Frasaco), and 1.15 N (±0.20) for the 3D‐printed typodont teeth. In the buccolingual direction, forces recorded were 1.04 N (±0.18), 2.28 N (±0.25), and 1.12 N (±0.17), for extracted, commercial and 3D‐printed respectively. In the occlusal direction, 1.44 N (±0.26), 3.71 N (±0.34), and 1.37 N (±0.17), for extracted, commercial and 3D‐printed respectively, were recorded. A one‐way ANOVA test with a Tukey post hoc test (*p* < 0.05) showed significant differences (*p *= 4.81 × 10^−8^) between cutting extracted and commercial teeth in all directions, with commercial teeth requiring more force. Significant differences (*p* = 3.79 × 10^−8^) were also found between commercial and 3D‐printed typodont teeth, with commercial teeth requiring more force. However, no significant difference (*p* = 0.53) was observed between extracted and 3D‐printed typodont teeth.

All 20 clinicians from Study 2 completed questionnaires after an average of 21 min of cutting and interacting with the provided typodont teeth (Frasaco and 3D‐printed).

**FIGURE 6:**
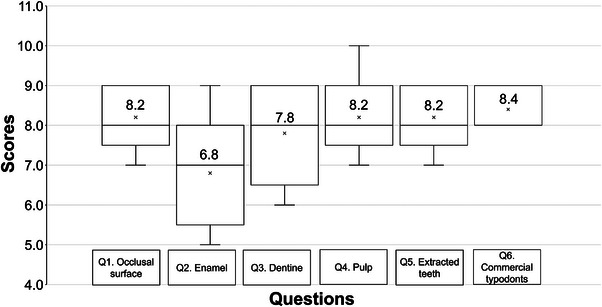
Dental educators’ response to the questions to the numerical response scale questions included in the questionnaire, rating the key features of the 3D‐printed typodont (i.e., occlusal surface, perception of cutting enamel, dentine and exposing the pulp). Mean values are given within the bow and whisker plot per question (marked “*”). Median values are given as a solid line within the plots.

In Question 1, participants rated the occlusal surface of the 3D‐printed typodont teeth compared to an extracted tooth with an average score of 8.2 (±0.84) out of 10 (Figure 6). This was the second‐highest response, indicating that participants ranked the occlusal surface highly, comparable to an extracted tooth.

In Questions 2 and 3, participants rated the likeness of drilling the printed enamel and dentine (respectively) compared to human extracted teeth. Enamel scored 6.8 (±1.48) and dentine scored 7.8 (±1.30) out of 10 (Figure 6). Clinicians found the 3D‐printed dentine to be a better representation of human extracted teeth, which was reflected in their answers to Questions 8 and 9. The responses between these questions were significantly different (*p* = 0.04), again suggesting that dentine was seen more favorably. Although scores were high, improvements can be made to ensure the materials more closely resemble natural tissues.

In Question 4, participants rated the likeness of exposing the pulp, with an average score of 8.2 (±1.10) out of 10 (Figure 6). However, six out of the eighty 3D‐printed typodont teeth did not contain a pulp chamber due to printing inconsistencies.

In Questions 5 and 6, participants rated the overall value of operative experience and training compared to human extracted teeth and commercial typodont teeth. The responses were 8.2 (±0.84) and 8.4 (±0.55), respectively (Figure 6). Question six had the highest response, indicating that clinicians believed the overall value was better with the printed typodont teeth compared to commercial typodont teeth, despite the enamel not matching the likeness of drilling natural tissue (Question two 6.8 ± 1.48). Analysis showed a significant difference (*p* = 0.04) between the responses for Questions 5 and 6.

Questions 8 and 9 asked participants about their least and most liked aspects of the model. For Question 8, recurring answers mentioned soft enamel, remnants of support structure (excess material) from the printing process on the occlusal surface (Figure 2A), and unrealistic colors. However, for Question 9, participants liked the use of different materials to differentiate structures, rated the likeness of drilling dentine as a highlight, and found 3D‐printed typodont teeth easier to cut, resembling natural teeth, unlike Frasaco teeth provided. These comments indicate limitations and strengths of the 3D‐printed typodont teeth, such as soft enamel and remnants of support structure, but also advantages in terms of likeness to natural teeth and ease of cutting.

Free‐text comments praised efforts to create realistic tooth likeness. Clinicians preferred natural color, appreciated the noticeable change in tactility during drilling from enamel to dentine, resembling human extracted teeth, and found it the most favorable and comparable to human teeth.

## 4 DISCUSSION

The studies aimed to assess both objective and subjective aspects of cavity preparation. Study 1 measured forces during cavity preparation, providing quantitative data. Study 2 gathered qualitative feedback from different clinicians to avoid bias. Separate groups ensured unbiased, comprehensive comparisons between objective measurements and subjective feedback. The clinicians participating in the study varied in their years of experience, as no specific inclusion criteria related to the level of experience were applied. This approach allowed for a broad representation of clinical perspectives. The results indicate differences between the typodonts evaluated. The aim of developing effective 3D‐printed typodont teeth is to replicate cutting responses for human extracted teeth. Critically, the structures and manufacture of the 3D‐printed typodonts will determine the results for Studies 1 and 2.

Enamel and dentine were printed separately and fixed together with additional enamel material; the oversizing of the enamel proved invaluable in fixing the materials together. However, from the XMT (Figure 2), evidence of large voids in the artificial enamel–dentine interface as well as settling of the CHAp on the surface of the dentine was observed. This settling is important as this shows the mixture was not homogenous, compared with the rest of the 3D‐printed typodont. The presence of voids within the enamel–dentine interface was linked to the curing of the additional material added. In Study 1, there were no apparent signs that the voids affected the force data being recorded. This may be due the nature of cutting, with clinicians removing a larger size of material than that of the voids (<300 µm), meaning the measurements were not sensitive enough to measure the voids. Cutting force data ranged from 1.12 to 1.37 N for the 3D‐printed typodont teeth, human extracted teeth ranged from 1.04 to 1.44 N, and commercially available artificial teeth ranged from 2.20 to 3.71 N. The data between the three groups show that the developed composites resemble that of human extracted teeth more closely than the commercial artificial teeth. Data collected by Cresswell‐Boyes et al.^15^ demonstrated multiple compositions that closely resembled that of extracted enamel and dentine in terms of required cutting force.

It was noted during Study 2 (questionnaire) that some of the 3D‐printed typodont teeth did not have a fully formed pulp chamber or in some cases a complete absence of one as shown in Figure 2. This absence of designed voids is a common problem when manufacturing parts using stereolithography/direct light processing and selective laser sintering 3D printing technologies. Uncured or unsintered material can become trapped within the printed part, which can cause issues especially when trying to recreate a product with internal anatomy. This factor was evident in the study, with only some of the typodont teeth possessing a pulp chamber and root canal. A future design solution would be to enlarge the pulp chamber at the image manipulation stage of design, allowing for this trapped material to be evacuated effectively during the washing stage of production; a suggestion that is currently being researched in further work. Despite this, however, the typodont teeth that did contain a pulp cavity were rated highly within the study, with educators scoring an average of 8.2 (±1.09).

When evaluating the clinical dental educators’ opinions of the developed 3D‐printed teeth (Study 2), Frasaco teeth were chosen as a comparison due to their use within the Queen Mary University of London, as well as their force required to cut being closer to natural teeth than the other commercial typodont teeth that were tested.^15^ The 3D‐printed typodont was manufactured to look like the Frasaco tooth. The dental educators stated that the 3D‐printed tooth was easier to cut, and when likened to human extracted teeth, the 3D‐printed tooth ranked highly against commercial teeth. This vast difference in haptic perception was most likely due to the amount of force needed to cut the tooth, as previously established in Cresswell‐Boyes et al.^15^ With extracted enamel requiring 0.31 N (±0.12), Frasaco enamel requiring 0.69 N (±0.21), and the 3D‐printed enamel (5 wt.% CHAp) requiring 0.36 N (±0.03), this difference in force is reflected in the educators’ perception on ease of cutting as well as the haptic perception between the human extracted teeth and the 3D‐printed teeth. For this study, 5 wt.% CHAp (dentine) and 25 wt.% HAp (enamel) was chosen due to both lower manufacturing costs, in terms of reduced particle contents and similarity in drilling experience to human extracted teeth 0.47 N (±0.18) and 0.31 N (±0.06) for the dentine and enamel, respectively. However, answers to Question 8, suggesting the enamel was “too soft” when cutting, were reflected in the response to Question 2, which received a response of 6.8 (±1.5) out of 10. This was the lowest response when compared with the other values, suggesting further work is required in recreating enamel. Thus, despite similar cutting forces extracted, there is still a disconnect between haptic perception and the forces recorded.

Overall, 100% of the educators said they would use the 3D‐printed typodont teeth again in future simulated practices. However, due to the small sample size used in the feedback, it is difficult to conclude a final suggestion that these 3D‐printed typodont teeth could be used as a replacement for Frasaco teeth; more feedback, as well as comparisons to other commercially available artificial teeth, are required before firm conclusions can be drawn. A larger sample size would also allow for the reliability and validity of the questionnaire to be tested.^27^ As discussed in Cresswell‐Boyes et al.,^15^ the force experiments carried out established that more force was required to cut commercial typodont teeth compared to extracted ones. Force of 0.31 N was needed to cut extracted enamel, whereas more than double the force was required to cut the lowest force of commercial enamel tested, 0.64 N (Fabrica de Sorrisos), with Frasaco recording the second closest force with 0.69 N. Moreover, the force more than tripled when compared to the highest force of 1.13 N (One Dental) recorded for typodont teeth. This discrepancy in forces may explain why undergraduate students dislike the use of typodont teeth.^14^, ^16^ In terms of dentine, Frasaco recorded a force of 0.64 N, the closest match from the commercial typodonts tested compared to extracted dentine, 0.49 N. The similar forces seen in the enamel and dentine of the Frasaco typodont teeth were due to their uniform composition and solid structure (no differentiation between the two tissues). Future studies would focus on larger sample sizes although initial results show a promising perception from educators when using the developed 3D‐printed teeth. Further refinements to the manufacturing process were identified here to ensure the 3D‐printed typodont teeth are equal, that is, each typodont includes a pulp chamber and enamel esthetically similar to extracted tissues. Furthermore, future studies could also investigate the interaction of commercially used bonding agents and dental cements with 3D‐printed typodont teeth.

## 5 CONCLUSION

This study aimed to build on previously published work, to explore the link between haptic perception and cutting forces. The 3D‐printed typodont closely matched the forces that were measured on human extracted teeth. The 3D‐printed typodont teeth were rated higher than that of the commercial teeth (Frasaco) in terms of overall operative experience, suggesting that the participants in this study would prefer to use the printed typodont teeth in the future over the commercial product. The enamel material used was noted as requiring improvement in terms of haptic response and aesthetics. Future studies should investigate improving the aesthetic appearance of the materials such as employing a ceramic glaze as part of the typodont manufacturing process.

## AUTHOR CONTRIBUTIONS

**Alexander J. Cresswell‐Boyes**: Investigation; data curation; visualization; writing—review and editing. **Graham R. Davis**: Conceptualization; supervision; writing—review and editing. **Asa H. Barber**: Supervision; writing—review and editing. **Mahentha Krishnamoorthy**: Supervision; writing—review and editing. **Swati R. Nehete**: Conceptualization; data curation; writing—review and editing.

## CONFLICT OF INTEREST STATEMENT

The authors declare no conflicts of interest.
